# Individualization of Treatment with Gentamicin in Neonates Based on Drug Concentration in The Blood Serum

**DOI:** 10.34763/devperiodmed.20192301.2127

**Published:** 2019-04-08

**Authors:** Magdalena Hurkacz, Joanna Monika Nowakowska, Dorota Paluszyńska, Barbara Królak-Olejnik

**Affiliations:** 1 Department of Clinical Pharmacology, Wrocław Medical University, Wrocław, Poland; 2 Department and Clinic of Neonatology, Wrocław Medical University, Wrocław, Poland

**Keywords:** gentamicin, newborns, pharmacokinetics, therapeutic drug monitoring, gentamycyna, noworodki, farmakokinetyka, terapia monitorowana

## Abstract

**Aim:**

To evaluate how useful it is to make measurements of gentamicin concentrations in newborns' b/ood in order to optimize antibiotic therapy.

**Material and methods:**

73 newborns empirically treated with gentamicin, in doses consistent with the Neofax^®^ guidelines. There were 152 measurements of maximum and minimum serum gentamicin concentrations. Samples were determined based on the chemiluminescence technique on the Siemens Advia Centaur analyzer. The concentrations of gentamicin that were measured were compared with various therapeutic ranges used in the literature.

**Results:**

According to the standards adopted in the University Hospital in Wrocław, the maximum concentration was reached in 38.16% of all the chi/dren, white the minimum in 26.32%. In other chi/dren the concentrations were below or above the therapeutic range. According to the Neofax^®^ guidelines, the intended maximum concentration was observed in 71.05% of the newborns, and the minimum in 32.89%. The minimum concentration of <2 mg/L was found in 93.42% of the newborns, white >2 mg/L was determined in 33.33%, despite a 48-hour dosing interval. These were premature ba bies (<28th week of gestational age) and 55.56% of them reached a maximum concentration of 5-12 mg/L.

There was no significant correlation between maximum or minimum concentration and gestational age or body weight.

**Conclusions:**

1. The dosage of gentamicin in newborns according to the Neofax^®^ recommendations does not ensure achieving the intended serum antibiotic concentrations. 2. In order to optimize gentamicin therapy in newborns it is necessary to individualize the dose based on measurements of drug concentrations in the blood and pharmacokinetic calculations.

## Streszczenie

**Cel:**

Ocena przydatności pomiarów stężeń gentamycyny we krwi w celu optymalizacji antybiotykoterapii u noworodków.

**Materiał i metody:**

Do badania włączono 73 noworodki leczone gentamycyną. Antybiotyk był podawany empirycznie i dawkowany zgodnie z wytycznymi Neofax^®^. U dzieci wykonano 152 pomiary stężeń gentamycyny w surowicy **–** maksymalnych oraz minimalnych. Próbki oznaczono za pomocą techniki chemiluminescencji na analizatorze Siemens Advia Centaur. Porównano stężenia gentamycyny oznaczone u dzieci z zakresami terapeutycznymi podawanymi w piśmiennictwie i normami ujętymi w zaleceniach terapeutycznych.

**Wyniki:**

Według norm przyjętych w Uniwersyteckim Szpitalu Klinicznym pożądane stężenie maksymalne leku 5-10 mg/l osiągnięto u 38, 16% dzieci, a stężenie minimalne w zakresie 1-2 mg/l –u 26,32% dzieci. Według wytycznych Neofax* pożądane stężenie maksymalne 5-12 mg/l zaobserwowano u 71,05% noworodków, a stężenie minimalne wynoszące 0,5-1 mg/l – u 32,89% dzieci. Stężenie minimalne <2 mg/l stwierdzono u 93,42 % noworodków, natomiast >2 mg/l oznaczono u 33,33% dzieci, mimo 48 godzinnego przedziału dawkowania. Były to wcześniaki urodzone przed 28 tygodniem wieku płodowego. 55,56% z nich osiągnęło stężenie maksymalne 5-12 mg/l.

Nie wykazano istotnej korelacji pomiędzy wiekiem płodowym a stężeniem maksymalnym oraz minimalnym, masą ciała a stężeniem maksymalnym i minimalnym.

**Wnioski:**

1. Dawkowanie gentamycyny u noworodków zgodnie z zaleceniami podanymi w Neofax* nie zapewnia osiągnięcia zamierzonych stężeń antybiotyku w surowicy. 2. W celu optymalizacji terapii gentamycyną u noworodków konieczny jest indywidualny dobór dawkowania na podstawie pomiarów stężeń leku we krwi i obliczeń farmakokinetycznych.

## Introduction

Newborns are a special group of patients. The pharmacokinetic and pharmacodynamic processes occurring in their organisms are different from those observed in other groups. Moreover, they show intra-population differences [1, 2]. Standard dosing of medications, in accordance with the adopted schemes for neonates, does not guarantee an effective and safe treatment process. This particularly applies to drugs with a narrow therapeutic index, including aminoglycosides. Slight changes in their serum concentrations may result in the lack of therapeutic effect or the occurrence of adverse effects, mainly related to ototoxicity and nephrotoxicity. If a bacterial infection is suspected in a newborn gentamicin is used as a first-line treatment in combination with ampicillin [3] . Due to the immaturity of the immune system in such patients, the most common infections are general ones [4] . Mortality rates, especially in the group of extremely immature, prematurely bom newborns, reaches even 20-50% in the course of sepsis [5]. Undertaking effective but concurrently safe treatment is possible for example with Therapeutic Drug Monitoring (TDM) – based therapy [6, 7, 8, 9]. Another parameter considered most useful in the optimization of aminoglycoside therapy is the ratio between drug maximum concentration in the blood at steady state (Cmax, peak level) to the Minimum Inhibitory Concentration (MIC). For gentamicin, the Cmax/MIC coefficient values in the range 8-10 are considered therapeutic [10, 11]. However, in many hospitals, common MIC determination is not practiced, therefore it is preferable to develop an accessible and useful method of individualizing antibiotic therapy in neonatology.

Unfortunately, despite the greater availability of biochemical analyses in health care facilities, no clear guidelines have been developed so far, specifying the recommended range of gentamicin serum concentrations in newborns. The maximum concentration (C ) of 5 to 12 mg/ L and the minimum concentration (Cmin, trough level) below 2 mg/L is most often considered therapeutic [10, 12, 13]. In the literature, few dosing regimens of gentamicin in neonates are available. Most often they are taking into account the newborns gestational age and body weight. Despite therapeutic recommendations, antibiotic concentrations and clinical efficacy are not achieved in all infants [14, 15, 16].

The aim of the study was to assess the usefulness of measuring serum gentamicin concentrations for the optimization of neonatal therapies. The evaluation of selected pharmacokinetic parameters of this drug will be the subject of another study.

## Material and methods

### The characteristics of patients

The study group consisted of 73 newborns (including 62 premature infants – 84.93%) treated in the Department of Neonatology at the University Hospital in Wroclaw due to bacterial infection. In the newborns tested, 152 measurements of maximum and minimum gentamicin serum concentrations at steady-state were performed. The neonates were treated in the period from October 2015 to December 2016. The study received permission of the Wroclaw Medical University Bioethics Committee No. KB-489/2013. Among the neonates examined there were 39 boys and 34 girls. The average gestational age (GA) was 33 weeks (tab. I).

**Table I:** Gestational age of newborns treated with gentamicin. Tabela I. Wiek płodowy noworodków leczonych gentamycyną.

Study group	Gestational age [weeks] *Wiek płodowy [tygodnie]*		
*Badana grupa*	25-28 29-32	33-36	37-41
Newborns [*Noworodki* number/percentage] *[liczba/odsetek]*	9/12.33 25/34.25	28/38.35	11/15.07

31 infants were born with moderately low birth weight (MLBW) in the range of 1500-2499 g, 12 children had very low birth weight (VLBW) of 1000-1499 g, and 6 children had an extremely low birth weight (ELBW) in the range of 500-999 g.

In 49 patients, sterile microbial culture was found, whereas in 24 the culture was positive. In 7 newborns, infections caused by several pathogens were found simultaneously, and in one child bacterial infection was accompanied by fungal infection caused by *Candida parapsilosis* (in this case, additional antifungal treatment was used).

All the newborns received a combination therapy with gentamicin and ampicillin as empirical treatment. Only one newborn was introduced on the 8th day of life. In the other infants, antibiotic therapy was implemented on the third day of life at the latest. Clinical improvement was observed in those children with a confirmed bacterial infection in whom the concentrations were consistent with the recommendations or the treatment was modified to the therapeutic range.

Antibiotic dosage was adjusted in accordance with the guidelines contained in Neofax^®^ and depended on the gestational age of the newborns. [17] Gentamicin was administered as a one-hour intravenous infusion (tab. II).

**Table II:** Dosing regimen of gentamicin in newborns. Tabela II. Schemat dawkowania gentamycyny u noworodków.

Gestational age [weeks] *Wiek płodowy [tygodnie]*	Dose [mg/kg] *Dawka [mg/kg]*	Dosing range [hours] *Przedział dawkowania [godziny]*
<29*	5	48
30 do 33	4.5	48
34 do 37	4	36
>38	4	24

*or clinically significant hypoxia, PDA or treatment with indomethacin/lub istotnie kliniczne niedotlenienie, PDA lub leczenie indometacyną

### Sampling and measurement of drug concentration

After reaching gentamicin steady state, two ~1 ml venous blood samples were collected into tubes with a separator – the first one 30 minutes after the administration of gentamicin (maximal concentration – C ), the second one 30 min before the administration of the next dose (minimum concentration: C_min_), in order to determine the concentration of the drug from each newborn. The concentration of the drug was measured in blood serum using a Siemens Advia Centaur analyzer by means of the chemiluminescence technique (Chemiluminescence immunoassay, CLIA). In the solid phase there are a limited number of mouse monoclonal antibodies against gentamicin molecules conjugated to paramagnetic particles. The gentamicin molecules derived from the patient’s blood compete with the antibodies to the acridine ester molecules in the liquid phase. The procedure is automated [18].

### Statistical analyses

Mean standard deviation, the median, the interval between the first and third quarter (IQR), minimum and maximum values were calculated for all the data and pharmacokinetic parameters. The normality of the distribution was assessed using the Shapiro-Wilk test. The hypothesis about the normality of distribution was rejected, therefore non-parametric tests were used in the next stage. Relationships between variables were calculated using Spearman’s correlation. In all the tests, p <0.05 was assumed as the level of significance. The statistical analysis was performed using the Statistica 12.0 program.

## Results

**Fig. 1:**
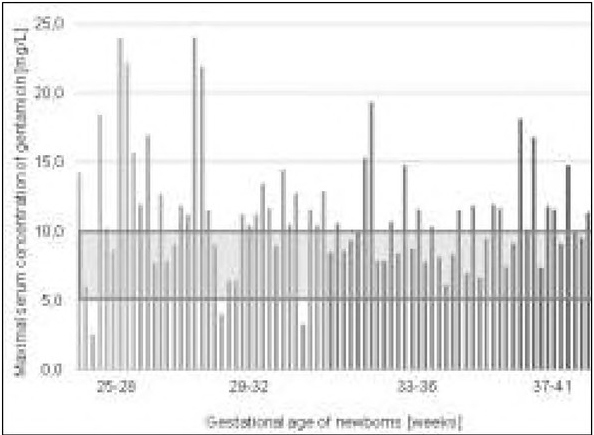
Maximum concentrations of gentamicin determined 30 minutes after antibiotic therapy (C_max_) in the serum of the newborns (the standard valid in the hospital was marked). Ryc. 1. Stężenia maksymalne gentamycyny oznaczone 30 minut po podaniu antybiotyku (C_max_) w surowicy badanych noworodków (zaznaczono normę obowiązującą w szpitalu).

**Fig. 2:**
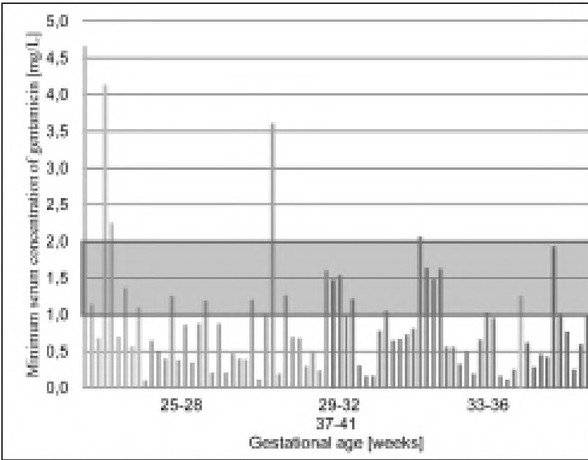
Minimum concentrations of gentamicin determined30 minutes before the next dose of antibiotic (C_min_) in the serum of the newborns (the standard valid in the hospital was marked). Ryc. 2. Stężenia minimalne gentamcyny oznaczone 30 minut przed podaniem kolejnej dawki antybiotyku (C_min_) w surowicy badanych noworodków (zaznaczono normę obowiązującą w szpitalu).

The maximum and minimum concentrations of gentamicin in the serum were taken as the results of the effectiveness and safety of the antibiotic therapy (Fig. 1 and 2).

The Spearman test evaluated the correlation between maximum and minimum concentrations of gentamicin in the serum, gestational age and the body weight of the newborns. There was no significant correlation between gestational age and C_max_ (R=-0.145, p=0.212) and C_min_ (R=-0.083, p=0.474). Correlations between body weight and C_max_ (R=-0.170, p=0.142) and C_min_ R=-0.019, p=0.871) were not statistically significant, either.

In 18 patients (24.66%) it was necessary to discontinue gentamicin and introduce another antibiotic in accordance with the antibiotic susceptibility test obtained. Only 4 patients received monotherapy, in the remaining ones gentamicin was replaced with more than one antibiotic.

**Comparison of the maximum and minimum concentrations of gentamicin determined in the newborns tested against the ranges recommended in the scientific literature**:

The literature assumes different reference ranges of maximum and minimum concentrations of gentamicin in serum. The study presented compares the *C* and C _min_ . values achieved in neonates treated with antibiotics according to the Neofax* guidelines with the therapeutic ranges proposed in the literature.

### Maximum concentration

In the laboratory of the University Hospital, C_max_ 5-10 mg/L was assumed for the therapeutic range and 5-12 mg/L was specified in the in Neofax^®^ guidelines [17]. In the literature available, these values are most often considered as therapeutic ranges [15,17]. According to Begg et al., C_max_ should be > 10 mg/L [16], and according to van Maarseveen et al. C_max_> 8 mg/L [19].

Taking the therapeutic ranges of gentamicin concentrations binding at the laboratory of the University Hospital in Wroclaw as a norm, correct C_max_ values were observed in 38.16% of the newborns tested, most often in infants born at 33-36 weeks of gestational age (57.14% of the population studied), least often – newborns at 25-28 weeks of gestational age (22.22%). The maximum concentration below 5 mg/L (subtherapeutic) was recorded in patients born before 32 weeks of gestational age (3.95% of all patients). Maximum concentrations above the hospital norm were observed in 57.9% of the population of neonates enrolled in the study. In accordance with the Neofax* guidelines, desirable m Cax values were observed in 71.05% of the newborns tested (most often born at GE 33-36 weeks, least frequently at the gestational age of 25-28 weeks, (tab. III). On the other hand, 25% of the newborns were placed above the therapeutic range for this norm.

**Table III:** Percentage of neonates subjected to testing who reached therapeutic ranges of maximum concentrations based on various standards specified in the literature. Tabela III. Odsetek badanych noworodków, u których osiqgnięto terapeutyczne zakresy stężeń maksymalnych na podstawie różnych norm określanych w piśmiennictwie.

Reference source *Źródło odniesienia*	Compared therapeutic range [mg/L] *Porównywany zakres terapeutyczny [mg/L]*	Percentage of newborns reaching the therapeutic range *Odsetek noworodków osiągających zakres terapeutyczny*
Hospital therapeutic range [21] *Norma szpitalna*	5-10	38.16%
Neofax [19]	5-12	71.05%
Begg [16]	>10	51.39%
van Maarseveen [19]	>8	68.49%

### Minimum concentrations

For the minimum gentamicin concentration, the standard in the hospital laboratory is 1-2 mg/L. Neofax*, which is optimal for values in the range of 0.5-1 mg/L [17]. In the literature analyzed, the minimum concentration which is less than or equal to 2 mg/L is usually considered as the reference [10, 15, 16] However, also C_min_ below 1 mg/L is considered as the reference value [6, 18]. Van Maarseveen et al. set the standard at C_m_ C_i_. _n_ below 0.5 mg/L [19].

Using the Neofax® standard, target C_min_ was achieved in 32.89% of the children examined (most often at the gestational age of 33-36 weeks, least often: 29-32 weeks). C_min_ values in the range of 1-2 mg/L were observed in 26.32% of the children, most often at the gestational age of 25-28 weeks (33.33%), and seldom at 29-32 weeks of gestational age (21.43%) (tab. IV).

**Table IV:** Percentage of neonates subjected to testing who reached therapeutic ranges of minimum concentrations based on various standards specified in the literature. Tabela IV. Odsetek badanych noworodków, u których osiągnięto terapeutyczne zakresy stężeń minimalnych na podstawie różnych norm określanych w piśmiennictwie.

Reference source *Źródło odniesienia*	Compared therapeutic range [mg/L] *Porównywany zakres terapeutyczny [mg/L]*	Percentage of newborns reaching the therapeutic range *Odsetek noworodków osiągających zakres terapeutyczny*
Hospital therapeutic range [21] *Norma szpitalna*	1-2	26.32%
Neofax [19]	0.5-1	32.89%
Begg [16]	<1	80.92%
van Maarseveen [19]	<0.5	38.35%

## Discussion

Therapy monitoring the concentration of the drug is gaining more and more importance in Poland, due to the greater availability of research in clinical practice. Its use is particularly important in the treatment of newborns whose pharmacokinetic processes differ from adults [20]. In addition, this group of patients is inhomogeneous. Depending on the gestational age of the newborn, differences in the values of pharmacokinetic parameters are observed.

Gentamicin is one of the aminoglycoside antibiotics that have bactericidal properties. Aminoglycosides act synergistically with cell wall synthesis inhibitors, e.g. β-lactam antibiotics, vancomycin [21, 22, 23]. During the treatment of newborns, large discrepancies in the values of pharmacokinetic parameters were observed. In a review work performed by Pacifici, the average biological half-life of gentamicin in newborns was between 4.9 and 4.6 h, the distribution coefficient 0.45-0.75 1 / kg, and clearance 0.53-1.72 ml/min/kg [2].

The most commonly recommended dose of gentamicin is 4-7 mg/kg body weight. Several different dosage guidelines are available, but these do not provide the intended levels for all children. Valitalo et al. performed a simulation, following four recommendations: Dutch, British, AAP and Neofax^®^. The highest percentage of expected results is achieved in the case of the Dutch guidelines. The maximum concentration in the range of 5-12 mg/L was obtained by 82% of patients, and the minimum <1 mg/L – 65%. The authors proposed their own dosing regimen in which C_max_ should be in the range of 5-12 mg/L and <0.5 mg/L. Based on these guidelines, the intended C_m ax_ was achieved in 82% of the newborns and in 93% [15]. Our own guidelines for gentamicin dosing in newborns are also suggested by other authors, taking into account various factors such as: the age and body weight of the newborn [16, 25], plasma creatinine concentration [27], creating a population model [25]. König et al. administered gentamicin at a dose of 4 mg/ kg body weight every 36 h in newborn fetuses <28 weeks and every 24 h in older newborns. In 10.2% of the patients the gentamicin minimum concentration exceeded 2 mg/ L [14]. Fonzo-Christe et al. proved that after the introduction of TDM recommendations in hospital, the percentage of measurements having reached the desired C . value below 1 mg/ L increased [6]. These recommendations consisted in determining the initiating dose based on their own schedule developed as an internal procedure, followed by measurement of the minimum and maximum concentration in each neonate. On this basis, after the calculation of pharmacokinetic parameters, the dosage was modified (the dose or dosing interval was changed). These calculations were made by a clinical pharmacist (or clinical pharmacologist). Testa et al. compared gentamicin concentrations in newborns treated with the standard dose of 2.5 mg/kg every 12 hours (28.6% of the children had normal maximum and minimum antibiotic concentrations) with neonates treated under TDM in which the dose and dose range depended on gestational age, body weight, the clinical status of the patient and concomitantly used drugs. Dosage was also modified if abnormal levels of gentamicin were found in a child. After this procedure, 68.1% of the children achieved the intended concentrations. The authors stated that without using TDM, patients would be exposed to the toxic effects of the antibiotic [25].

New dosage regimen recommendations are regularly published, but they still do not guarantee the achievement of the intended concentrations of gentamicin in all neonates. For this reason, in accordance with the Summary of Product Characteristics, it is recommended to conduct therapy monitored by the concentration of the drug in the blood [21].

Fourteen newborns of the gestational age between 25 and 41 weeks, participated in the study. Gentamicin was administered in empirical therapy and dosed according to the Neofax* guidelines [17]. The dosage regimen was adjusted according to the values of concentrations and the clinical state of the newborn, after consultation with the clinical pharmacologist. The pharmacokinetic parameters for each neonate were calculated and based on the desired effect; the dose or the range of dosages was changed. After reaching a new steady-state, the concentration of the drug in the serum of the neonate was monitored.

World data show that the mortality rates due to sepsis in the population of newborns vary between 20%-50% [5]. One newborn died in our study group (1.37%). In the neonatal population, sepsis confirmed by positive bacterial culture, was found in 24 newborns. In this group, the mortality rate was 4.1%, which indicates that the survival outcome was better than that stated in the literature. According to the authors, monitoring of pharmacotherapy using the concentration of drugs in blood measurements may have contributed to this condition.

## Conclusions

1. The dosage of gentamicin in newborns according to the recommendations provided by Neofax* does not ensure that the intended serum antibiotic concentrations have been reached, especially in relation to the value of minimum concentrations in newborns at the fetal age of 29-32 weeks.
2. Therapy monitored with gentamicin concentrations increases the effectiveness and safety of the treatment process.
3. The development of own prophylactic procedures for the dosage of gentamicin in neonates and the retrospective determination of optimal ranges of maximum and minimum concentrations of gentamicin in the hospital’s own population based on the available literature and clinical observations may contribute to an increase in efficacy and a reduction in the incidence and severity of the undesirable effects of aminoglycosides.
